# A Qualitative Evaluation of Two Electronic‐Rehabilitation Programmes for Managing Persistent Knee Pain

**DOI:** 10.1002/msc.70051

**Published:** 2025-01-09

**Authors:** Gretl A. McHugh, Elizabeth C. Lavender, Kim L. Bennell, Sarah R. Kingsbury, Philip G. Conaghan, Rana S. Hinman, Christine Comer, Mark Conner, Rachel K. Nelligan, Dawn Groves‐Williams

**Affiliations:** ^1^ School of Healthcare University of Leeds Leeds UK; ^2^ Department of Physiotherapy The University of Melbourne Centre for Health Exercise and Sports Medicine Melbourne Australia; ^3^ Leeds Institute of Rheumatic and Musculoskeletal Medicine University of Leeds Leeds UK; ^4^ NIHR Leeds Biomedical Research Centre Leeds UK; ^5^ Musculoskeletal and Rehabilitation Service Leeds Community Healthcare NHS Trust Leeds UK; ^6^ School of Psychology University of Leeds Leeds UK; ^7^ Clinical Trials Research Unit University of Leeds Leeds UK

**Keywords:** digital health, exercise, knee osteoarthritis, pain, qualitative study, rehabilitation

## Abstract

**Introduction:**

Persistent knee pain often due to knee osteoarthritis (OA) is a highly prevalent and disabling condition. Electronic‐rehabilitation (e‐rehab) programmes have the potential to support self‐management of knee OA. This study aimed to evaluate user engagement and acceptability of two e‐rehab programmes, Group e‐rehab, a remote physiotherapy‐led programme and My Knee UK, a self‐directed web‐based exercise programme.

**Methods:**

Descriptive qualitative study nested within a feasibility trial. In‐depth interviews were conducted remotely. Data were analysed using inductive thematic analysis.

**Results:**

Eighteen participants from the feasibility trial took part in the interviews, 10 who received Group e‐rehab and eight My Knee UK. Two key themes were engagement with exercise and impact of programme. Despite initial challenges with doing the exercises, most participants found both programmes acceptable and beneficial in improving symptoms and knowledge in managing their knee pain. Multiple factors contributed to motivation to exercise.

**Discussion:**

Understanding more about users' perception and acceptability of both programmes was important to ascertain, both from people who engaged and those who did not engage with the programmes, to make improvements for the future delivery of the e‐rehab programmes.

**Conclusion:**

Group e‐rehab and My Knee UK can support people to self‐manage their persistent knee pain due to knee OA. The e‐rehab programmes have the potential to improve health services by providing two new models of service delivery enabling more patients to receive support and training to equip them to effectively manage their knee OA.

## 1 Introduction

Persistent knee pain, often due to knee osteoarthritis (OA), is a highly prevalent musculoskeletal condition with a global prevalence of 16% (Cui et al. 2020). Knee OA is a disabling condition commonly associated with high levels of pain and reduced mobility (Ackerman et al. 2017; Zeni, Axe, and Snyder‐Mackler 2010; Peat, McCarney, and Croft 2001). Exercise programmes are successful approaches to help reduce pain and improve physical functioning in knee OA (Mo et al. 2023; Raposo, Ramos, and Lúcia Cruz 2021; Fransen et al. 2015). A systematic review concluded that patient education is more effective for improving pain and function in those with knee OA when combined with exercise therapy (Goff et al. 2021). The National Institute for Health and Care Excellence (NICE) recommends both exercise and education for managing symptoms of knee OA (NICE 2022).

Physiotherapists are key providers of exercise therapy, support and educational advice for individuals when managing knee OA. However, physiotherapy services and other community and primary care musculoskeletal services are overstretched. We have an ageing population and a rise in adult obesity, which are creating an increased demand for insufficiently resourced physiotherapy services in the National Health Service (NHS) (Centre for Ageing Better 2023; Office for Health Improvement and Disparities, 2023; The Chartered Society of Physiotherapy (CSP) 2024). Our overstretched NHS necessitates the need for alternative management strategies to be implemented. There is good evidence to support accessible and cost‐effective Internet‐delivered programmes for improving the management and treatment of health conditions (Rogers et al. 2017; Centre for Policy on Ageing 2014).

In Australia, there has been success in developing and evaluating electronic‐rehabilitation (e‐rehab) programmes for people with persistent knee pain, such as knee OA. One such programme involved home exercises prescribed by a physiotherapist via six individual videoconferencing consultations over 6 months combined with an automated pain‐coping skills training programme (Bennell et al. 2017). A randomised controlled trial showed that the programme enabled a bio‐psychosocial approach to managing persistent knee pain with clinical improvements demonstrated for both pain and function compared to online education. Another Australian programme is ‘My Knee Exercise’. This self‐directed programme comprised a web‐based education and 6‐month strengthening exercise component including videos together with automated text messages to support exercise behaviour change (Nelligan et al. 2019). This programme was effective for improving physical function and knee pain (Nelligan et al. 2021).

As part of this study, we adapted components of these previously tested Australian e‐rehab programmes for use in the UK (Grove‐Williams et al. 2023). These two e‐rehab programmes (Group e‐rehab and My Knee UK) then underwent feasibility testing. This paper reports on the findings of an embedded qualitative evaluation.

### 1.1 Aim of Study

The overall aim of the study was to evaluate the feasibility and acceptability of two e‐rehab programmes: group Internet‐delivered physiotherapist prescribed home exercise and interactive educational sessions (Group e‐rehab); and self‐directed web‐based home exercise programme with behaviour change messages (My Knee UK) for individuals with chronic knee pain. The qualitative study evaluated user engagement, experiences and acceptability of the two e‐rehab programmes. Feasibility trial acceptability issues were also explored but not reported in this paper.

## 2 Methods

### 2.1 Design

The study was a descriptive qualitative study (Doyle et al. 2020). This design is useful in providing a straightforward account of participants' experiences and perceptions (Sandelowski 2010). This design is also useful when qualitative research is embedded in intervention studies, as it enables an exploration of why an intervention worked or did not work (Doyle, Brady, and Byrne 2016). This study was nested within a non‐blinded randomised feasibility trial (Grove‐Williams et al. 2022). Ethical approval was obtained from the West of Scotland Research Ethics Committee 5 (REF: 20/WS/006). The reporting of the study is guided by the Consolidated Criteria for Reporting Qualitative Research (CONSORT) checklist (Tong, Sainsbury, and Craig 2007).

### 2.2 Sample

For the feasibility trial, we recruited 90 participants: 30 participants received their usual routine care and no additional intervention; 30 participants were randomly selected to receive ‘My Knee UK’; and 30 participants were randomly selected to receive Group e‐rehab. For the qualitative evaluation a sub‐sample of participants from the two intervention arms was purposively sampled based on gender, age, and intervention group. Our intended sample size was approximately 16–20 participants (∼8–10 from each intervention group); however, data collection continued until data adequacy was deemed to be achieved (Vasileiou et al. 2018).

### 2.3 Recruitment

Participants for the qualitative study were recruited from both intervention arms of the feasibility trial. Participants for the feasibility trial were recruited from primary and community care organisations, as per published recruitment procedure (Grove‐Williams et al. 2022). The inclusion criteria were:

- Adults ≥ 45 years;
- Knee pain >3 months and on most days of previous month;
- Knee pain during walking ≥ 4 on an 11‐point numerical rating scale;
- Activity‐related knee joint pain;
- Mobile phone with active email account and computer with internet access suitable for receiving and making video calls.

A sub‐sample of participants taking part in the feasibility study were invited to take part in an interview after completion of their intervention (either within 3 months or after 6 months of completion). Once consent forms were received, participants were contacted to arrange an interview. We varied the timing for the qualitative interviews due to practical reasons and to gain insight into continued engagement with the exercises.

### 2.4 Intervention

The process of adaption of the Australian programmes for use in the UK and the description of the interventions are published (Grove‐Williams et al. 2022 2023). In summary, the two UK e‐rehab programmes for evaluation were:

- Group e‐rehab, which involved a group‐based home exercise programme with Internet‐interactive education sessions, including a prescribed home exercise programme and physiotherapist‐monitored group exercise sessions. The exercise sessions were delivered via videoconferencing weekly to fortnightly over a period of 12 weeks. In addition to online group exercise classes, participants were asked to repeat the same exercise programme at home at least three times a week. Each group had between 4 and 7 participants per group. Information resources were provided via Microsoft Sway.
- My Knee UK was a 12‐week self‐directed web‐based home exercise programme comprising educational videos and resources, with exercise behaviour change support provided via text messaging.

Group e‐rehab and My Knee UK participants completed an identical lower limb strengthening home exercise programme comprised of the following:

- Weeks 1–6: sitting knee extension, side steps and calf raises;
- Weeks 7–12: the above plus mini (wall) squats and chair rises (sit to stands).

The recommended exercise dosage was 30 repetitions of each exercise (3 sets of 10). Guidance about regressing or progressing the exercises, based on individual strength and ability, was provided to all participants.

### 2.5 Data Collection

Demographic data on participants were collected for the feasibility trial at baseline. For the interviews, a topic guide was developed from previous research and the literature (Anderson et al. 2021) and was reviewed by a patient, public and involvement (PPI) member of the research team. Box 1 provides an overview of the topic guide. Interviews were led by an experienced qualitative researcher (EL) with support from a novice qualitative researcher (DGW), who had been involved in recruitment of participants and delivery of the feasibility trial. These interviews were conducted via video‐call or telephone between July 2022 and February 2023. All interviews were audio‐recorded and took between 25 and 60 min (average 40 min). Field notes were taken both during and after the interview. No non‐participants were present for the interviews. Eleven participants were interviewed within 3 months of completing the intervention (Group e‐rehab or My Knee UK), and seven were interviewed 6 months after completing the intervention programme.

**BOX 1 Topic guide for interview.**

1

- Experience of being in the feasibility study (recruitment, random allocation, questionnaire burden);
- Experience of completing the e‐rehabilitation programmes including usefulness and acceptability (also of educational component; impact of programme);
- Engagement and motivation;
- Perception of changes in ability to manage symptoms more effectively and impact of programme.

Specific E‐rehab programme areas:

*My Knee UK:*

- Exploration of web‐based home support, exercise levels, and the motivational text support.

*Group E‐Rehab:*

- Exploration of the consultation with the physiotherapist on‐line, including group interaction.
- Exploration of the support of peers on‐line, including informal peer support outside formal videoconference sessions.

### 2.6 Data Analysis

Interviews were transcribed verbatim by an independent agency, checked for accuracy and anonymised by the researchers. Data were analysed using inductive thematic analysis (Braun and Clark 2021). Inductive thematic analysis allows for a flexible approach and data‐driven exploration, without any pre‐existing theories or framework (Braun and Clark 2021). It is a useful approach when there are differing perspectives from participants, enabling similarities and differences to be explored (King 2004). Within data analysis, there was a shift from uninterpreted participant quotes to interpreted research findings, whilst remaining close to the data (Sandelowski 2010). Line‐by‐line coding was used. To assist with inter‐coding reliability (MacPhail et al. 2016), two researchers (G.A.M., E.C.L.) independently coded the data and where there were discrepancies, these were discussed and addressed. Microsoft Excel was used to organise data. There was no participant validation of the data. However, preliminary findings were discussed at a project advisory group meeting with two patient and public involvement (PPI) members with knee osteoarthritis, who contributed their insight and interpretation of the data.

## 3 Results

From the feasibility study, 13 participants receiving Group e‐rehab were invited to take part in the qualitative study, with 10 participants consenting and being interviewed. Of the participants receiving the My Knee UK programme, 15 were invited with eight participants consenting and being interviewed. A total of 18 participants took part in the qualitative study.

Table 1 provides demographic details of the participants. More females than males were interviewed in the Group e‐rehab sample, and the mean age was slightly higher in the My Knee UK sample. Over 50% of the total participants were employed at the time of the interview and all were of white ethnicity.

**TABLE 1:** Participant demographics.

	My Knee UK (*n* = 8)	Group e‐rehab (*n* = 10)
Age range (mean, SD)	53–67 (60.6 years, SD = 5)	49–75 (59.5 years, SD = 7)
Sex	4 Male	3 Male
	4 Female	7 Female
Ethnicity	7 White—British	10 White—British
	1 White—Irish	
Marital status	7 Married	7 Married
	1 Divorced	1 Divorced
		2 Single
Employment status		
Working	5	5
Retired	3	5
Duration of knee pain (months) (mean, SD)	10–300 (82 months, SD = 87)	10–480 (84 months, SD = 134)
Exercise confidence (mean, SD)^a^	5–10 (7, SD = 2)	5–10 (7, SD = 2)
Motivation to exercise (mean, SD)^b^	5–10 (8, SD = 2)	5–10 (8, SD = 2)

Abbreviation: SD = standard deviation.

^a^ Self‐reported confidence doing exercises to help with knee pain (10‐point Likert scale: 0 = no confidence to 10 being highly confident).

^b^ Self‐reported motivation for doing exercises to help with knee pain (10‐point Likert scale 0 = no motivation to 10 being highly motivated).

**FIGURE 1:**
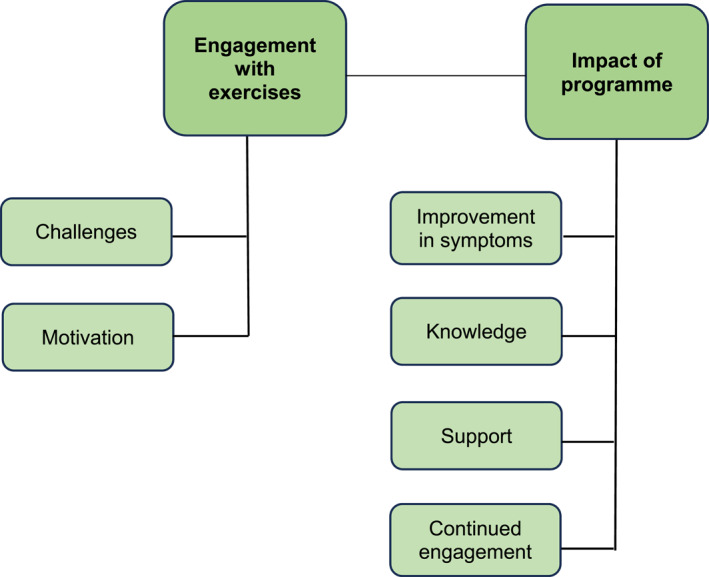
Overview of themes and sub‐themes.

The themes and sub‐themes derived from the data were constructed (Figure 1) and mapped across both intervention programmes. In this paper, the themes of ‘Engagement with exercises’ and ‘Impact of programme’ are presented.

### 3.1 Engagement With Exercises

For the majority of participants in both e‐rehab programmes, there was good engagement with the exercises, and they valued learning about the most appropriate exercises and gaining confidence to do the exercises. Participants said:

It was brilliant, all information step‐by‐step you follow… it was quite nice in pictures and explanation and each part of the exercise how you need to stay, your spine, your shoulders, your knee, yes it was quite good.

(Rufus, 62 years, My Knee UK)

I didn't realise how exercise could help in the way it did, so, yes, I'm definitely very pleased that I went on the exercise, very, on the scheme, because it's given me a lot more confidence that I'm not just going to roll over and say, well, okay, I'll have a new knee, then, please.

(Roger, 63 years, Group e‐rehab)

The My Knee UK participants reported that it took a few weeks to do the exercises before seeing some improvement in symptoms. Having different exercises and building the exercises into daily life helped with engagement. As one participant explained:

Once you’d done it a couple of times, it was alright and then you moved on to the next stage and do that a couple of times and it’s alright. As long as you’re the sort of person who, who can get into a regular routine. I mean, it is, it’s sometimes difficult for people to be in a routine. I just built into a day, my day’s routine.

(Alan, 67 years, My Knee UK)

Within the theme of engagement with exercises, sub‐themes of motivation and challenges arose.

#### 3.1.1 Motivation

Participants' self‐reported motivation for doing exercises to help with knee pain was relatively high prior to starting the programmes (Table 1). Multiple factors contributed to participants' motivation to exercise and included: increasing confidence, the convenience of home‐based exercise, realistic time for completing the exercises, and text reminders. In terms of confidence, having the support either through watching the exercises on the My Knee UK website or watching the physiotherapist do the exercises remotely for Group e‐rehab appeared to increase participants' motivation.

I found the whole thing positive and good for spurring me on. It was kind of really supporting the way I felt about the things I wanted to do and gave me an immense amount of confidence that exercise was the right thing….. that I should be pushing and strengthening and that this in the long‐term was what I needed and this was what was going to help me.

(Clare, 64 years, My Knee UK)

When you’ve got somebody [physio] there who quite clearly was an expert and has a way of motivating you to do it and encouraging you and showing you the right techniques….. the fact that you go in there and that you’re getting feedback there and then of how to improve your technique.

(Stuart, 49 years, Group e‐rehab)

Participants found the My Knee UK programme more convenient than Group e‐rehab due to its self‐directed nature and being able to exercise at a time of their own choosing. However, without the set time and group involvement, some participants found it harder to motivate themselves to exercise.

A distinct feature of My Knee UK compared to Group e‐rehab was that daily motivational texts were sent to participants asking whether they had completed their exercises. A participant explained:

I was getting those pings, and it was asking me, have you done your exercises today, so it makes you, you feel guilty if you haven’t so then you make more of an effort. I think, I know that at the end of the day it’s about my knee and it’s about my mobility moving forwards—and that should be enough to motivate me, but sometimes I think just having that, knowing that there’s somebody there that’s sort of pushing you on I think that helps.

(Carol, 61 years, My Knee UK)

For those who participated in Group e‐rehab, the motivational element was reported as having support from a physiotherapist and seeing the benefits which the exercises were having on their own (and others') symptoms.

That was extremely beneficial, that he/she was very much coaching us on the proper techniques as well, not just doing the exercises, but the proper techniques of doing it, ….. it was just the little things that he/she added to it that made a big difference. ..I had struggled with a lot of pain in my knees and that I was starting to see benefits from doing it, amongst the other exercises that I was doing as well, so I was starting to see the benefits from it, so my motivation was, you know, doing what I can to relieve some of the discomfort that I have in my knees.

(Stuart, 49 years, Group e‐rehab)

For each participant, the motivation needed to do the exercises was different. Some participants required the structure and contact with the physiotherapist to continue exercises, whereas others felt encouraged to practice exercises regularly because they noticed an improvement in symptoms.

#### 3.1.2 Challenges

All participants from each programme found one or two of the exercises difficult to do; however, this only appeared to affect engaging with the exercise for a minority of participants. Some reported that it took a couple of weeks to get used to doing the exercises. Challenges with the exercises were highlighted more by Group e‐rehab participants compared with My Knee UK participants. However, all participants felt that they could work at their own pace, gradually building up the frequency and duration of exercise sessions. One participant said:

You did what you could do, so if you could only do, say like the calf stretches, they were tough going, so they were probably the hardest ones to build up, but you just again you just did it at your own pace. So if you couldn’t do it, you didn’t do it, you did what you could.

(Isabel, 67 years, Group e‐rehab)

Some participants found it helpful to have visual cues, either watching the physiotherapist doing the exercises in Group e‐rehab; or watching the exercise video in My Knee UK. The instructions on how many repetitions for a specific exercise and how many times per day to practice the exercises were clear, and this helped participants work towards achieving their exercise goal.

### 3.2 Impact of Programme

Variable impact of the programme was another key theme described in terms of participants' perceived benefits of doing the programme. All My Knee UK participants and eight of the 10 Group e‐rehab participants found the programme to be beneficial overall. Four sub‐themes were constructed: improvement in symptoms, improvement in knowledge, feeling supported, and continued engagement.

#### 3.2.1 Improvement in Symptoms

Several participants reported an improvement in their symptoms, such as reduced pain, better mobility and gaining strength in the knee, enabling some to return to their regular activities. One My Knee UK participant said

I’m certainly finding that I have less pain and I can do far more. …It’s improved the muscle quality around my knee, so it’s supporting that knee joint.

(Alan, 67 years, My Knee UK)

Similarly, Group e‐rehab participants said:

I found the exercise, the group programme, again I found that good and I liked the exercises because I think they’ve definitely made a difference with me, I think there’s been some improvement. One of the things I’ve notice was that sometimes what I find is when I’m out and about, which can be a bit scary sometimes, me knee had actually went to give, since I’ve actually been doing the exercises, I’m actually getting less of that and I would say generally it feels a bit stronger.

(Janice, 62 years, Group e‐rehab)

I mean I found it incredibly satisfactory, it helped me enormously, evidence from the discussion with the consultant, it’s meant that I can now do an awful lot of things I couldn’t do, I still have pain, I still can’t sit for any length of time, I have to be careful when I move my knee if I’m sitting down because the knee locks into place but if I’m standing and it’s the effect of the improvement in my thighs and calf muscles they are enabling me to move around and do things that perhaps I wouldn’t have been able to do. I’m no longer having to use a stick.

(Edward, 75 years, Group e‐rehab)

#### 3.2.2 Knowledge

Both e‐rehab programmes included a range of resources and information. Most participants accessed these resources to support self‐management of their knee OA, commonly at the start of the programme. One participant who accessed a video about pain management on the My Knee UK programme said:

One of the little talks was about pain management and how you don’t really have to have a knee replacement, you know you can manage it yourself and that to, sort of like set me off when I listened to that particular clip if you know what I mean, and I thought you know that’s what I wanted you know, I don’t really want to go to having a knee replaced if I can avoid it.

(Joan, 67 years, My Knee UK)

Participants reported discovering new topics about self‐managing their knee OA. An example of this was learning more about living with OA and pacing.

I found those very useful indeed those educational videos, because I’d never really looked up arthritis or anything to do with pain in the past or anything really. I’d not really thought about it in those ways and so I did find those useful. You know, the idea of like managing your activity so it became not all one day and then nothing the next and that sort of thing.

(Helen, 56 years, Group e‐rehab)

The majority of participants from both programmes stated that the information provided was valuable and improved their knowledge of knee pain. However, a minority felt that they already had the knowledge to manage their knee OA. Some participants reported that they needed to be selective about the resources they looked at due to time pressures and often just focused on specific issues most relevant to them and the symptoms they were experiencing. A few participants enrolled in Group e‐rehab felt that the physiotherapists could have provided more linkage to the educational/information components of the programme, and this might have encouraged them to look at the information more regularly.

#### 3.2.3 Support

For participants who received Group e‐rehab, the support from the physiotherapist was seen as very valuable. However, there is a cost involved in having physiotherapist‐monitored group exercise sessions, which will be reported in the results of the feasibility trial.

There were few participants who could not always attend the remote sessions due to work commitments, highlighting the need to provide evening sessions. There were two of the eight My Knee UK participants who would have liked to receive physiotherapy input. Group e‐rehab participants cited being part of a group as being valuable and being able to connect with others with a similar condition. Participants said:

I’ve not done any online exercise stuff in a group before, I suppose it was just nice to be able to connect with people in a similar situation to me.

(Shirley, 50 years, Group e‐rehab)

However, most participants said that as the Group e‐rehab programme was of limited duration, it did not offer the ongoing group support, which for some would have been valuable. The My Knee UK programme did not have a personal contact; however, if participants experienced any difficulties while being part of the research study, they could contact a named physiotherapist for additional support.

Not all the participants highlighted the need for support. Two participants from My Knee UK indicated that they preferred doing exercises by themselves, finding it hard to schedule exercise with others and enjoyed the solitude. One participant said:

To be fair I probably like doing solitary things, so it was you know quite nice to have you know like half‐an‐hour or so, however long the actual exercises took to myself.

(Joan, 67 years, My Knee UK)

#### 3.2.4 Continued Engagement

Maintaining and sustaining exercise programmes are often an issue. Participants in this study varied in their continuation of the e‐rehab programmes. Those who continued did so because of the improvement in symptoms and mobility. Other participants reported to now have the knowledge to manage if symptoms became worse.

I found the whole thing very motivating and really want to keep doing it; and I quite often If I’ve not been to the gym or for some reason I’ve been too busy to go, then I’ll quite often go back and do the exercises if nothing else, if I’ve not been able to go anywhere.

(Isabel, 67 years, Group e‐rehab)

Some participants continued to do the exercises by building them into their daily routine.

Since doing My Knee UK programme I’m probably doing more of the exercises what have been on the programme, like I say I still do, I do them at work now.

(Mark, 55 years, My Knee UK)

However, others lost motivation once the research project finished and without the obligation to engage with the programme, there was a lack of interest in continuing to exercise.

I’ve finished the programme now, they won’t be bothered about me and I’ll chuck everything away. So my motivation’s been slightly down in the last week or so, or couple of weeks.

(Sheila, 60 years, Group e‐rehab)

A minority of participants perceived the exercises as not beneficial to them and this contributed to their lack of continued engagement. One participant said:

When I first started doing the exercises it all felt worse and it all felt more uncomfortable and then it started to get easier. If I’m honest I didn’t notice a massive improvement…. I think if I’d have noticed a lot of difference, I might’ve been better at continuing the exercises after the study was over… I probably felt a little bit disheartened and probably another reason why I didn’t carry on with the exercises. I wish I had.

(Maria, 58 years, Group e‐rehab)

Participants not seeing any noticeable improvement with symptoms became disengaged and didn't continue the exercise programme compared to those who benefitted.

## 4 Discussion

This qualitative study, nested within a randomised feasibility trial, has enabled an in‐depth view of participants' perception of two e‐rehab exercise programmes for persistent knee pain. Understanding more about user engagement and acceptability of both programmes was important to ascertain both from people who engaged and those who did not engage with their programme.

Digital interventions have been found to promote physical activity (Stockwell et al. 2019). However, personalisation and tailoring of digital interventions, such as choice of exercise intensity, are important components and would help facilitate engagement with digital exercise programmes (Mason et al. 2024). Both e‐rehab programmes had a degree of personalisation, with My Knee UK having an exercise intensity feature and Group e‐rehab being physiotherapy‐led, enabling a more personalised approach to exercise. Participants in this study valued being able to go at their own pace with these programmes. A qualitative evaluation of a UK digital intervention to improve physical activity in people with musculoskeletal conditions also found that personalisation is the key, and these interventions are not ‘one size‐fits‐all’ (Webb, Allison, and Mprah 2024).

### 4.1 Engagement With Exercises

Participants engaged with the exercises for both programmes and overall found the programmes to be useful and acceptable. Engagement may have been enhanced due to participants' experience with remote and video consultations for healthcare during the COVID‐19 pandemic. Motivation was a key element and monitoring via text messaging for My Knee UK was found to be beneficial. An umbrella systematic review found evidence supporting the value of integrative text message interventions, specifically in promoting adherence (Hall, Cole‐Lewis, and Bernhardt 2015). Those following Group e‐rehab had remote consultations with the physiotherapist, which was perceived as a motivating element. A qualitative study found monitoring of exercises by peers and instructors and seeing the personal benefits of exercises to be of value (Ledingham et al. 2019). These factors were similar in our study and contributed to participants being more engaged in doing the exercises.

With any exercise intervention, there are always some participants who feel they are benefitting and do not continue to engage with the exercises. In this study, a minority of participants did not engage in the exercises due to a perceived lack of benefit from doing them. Similarly, Ledingham et al. (2019) found that those with knee OA who had low adherence were ambivalent about the benefits of exercise. A qualitative study comparing responders and non‐responders to an exercise and physical activity intervention found that non‐responders acknowledged that their adherence to this intervention was poor but believed other factors such as life events and body weight contributed to this outcome (Hinman et al. 2023). This study found that work commitments influenced attendance at Group e‐rehab sessions, leading to poorer adherence to this programme.

Building a person's confidence to undertake exercise was a factor which promoted both motivation and engagement with the exercises. This was apparent with Group e‐rehab, where the physiotherapist supported and built‐up the groups' confidence during the sessions. Nelligan et al. (2020) found that having a ‘human connector’ is important for digital interventions and is a limitation for self‐directed programmes. Awareness of the time and cost of providing physiotherapist‐led exercise sessions need to be factored in for Group e‐rehab and whether a physiotherapist assistant could provide this remote exercise support would be useful to explore. The feasibility trial results will provide an estimation of the costs of delivering and the resources required for both e‐rehab programmes which need to be considered prior to implementing any new models of care for the management of knee OA.

### 4.2 Impact of Programme

Overall, all participants in the My Knee UK programme and eight of the 10 participants in the Group e‐rehab reported benefits particularly in terms of knowledge and improvement in symptoms. Having both education and exercise components was viewed as beneficial. Evidence has shown that patient education combined with exercise therapy is more effective for managing the symptoms of knee OA (Goff et al. 2021). The known barriers to physical activity participation in people with knee OA include lack of knowledge about the benefits and the fear that exercise will exacerbate pain and damage the joint (Dobson et al. 2016; Holden et al. 2012). Both programmes included an educational element that aimed to reduce misconceptions about OA and exercise and reinforce the benefits and safety of exercise. Participants reported that their knowledge increased and that they understood more about their condition and what exercise and other self‐management strategies would help.

Maintaining and sustaining exercise programmes are often an issue with a multitude of factors influencing non‐adherence to exercise (Marks and Allegrante 2005). It was encouraging that for both programmes, some participants had either continued to do the exercises or resumed a previous exercise activity. By interviewing some participants several months after the intervention had finished, we were able to ascertain their continued engagement with exercising.

E‐rehabilitation programmes may not suit everyone with managing their persistent knee pain and it is important not to exclude people with limited digital literacy. However, what this study has shown is that there is potential value for patients in providing alternative models of care for self‐managing persistent knee pain due to osteoarthritis. Indeed, recent research has shown that physiotherapist consultations via videoconferencing for the delivery of exercise‐based knee OA are non‐inferior to traditional in‐person care models (Hinman et al. 2024).

This study had a number of limitations. We were unable to interview participants at a consistent time point post‐intervention due to pragmatic reasons. Interviews were therefore undertaken either within 3 months or between six and 9 months after programme completion by participants. Consequently, individual recollections of the programme did vary. Although purposive sampling was used, more females than males took part in Group e‐rehab. There was poor ethnic diversity of participants with all being of white ethnicity. Despite steps to increase diversity, this was unsuccessful, and alternative strategies need to be developed for future research. Respondent verification was not undertaken with participants, which may have strengthened the trustworthiness of the data; however, two researchers were involved in data coding and analysis, and PPI members provided their perspectives on the data.

## 5 Conclusion

User engagement and experience of two e‐rehab programmes adapted for people in the UK have been investigated. Group e‐rehab and My Knee UK can support people to self‐manage their persistent knee pain due to OA. These e‐rehab programmes have the potential to improve health services by providing two new models of service delivery, enabling more patients to receive support and training to equip them to more effectively manage their knee pain. This qualitative evaluation provided an in‐depth account of how these e‐rehab programmes were perceived by users, and the challenges to engagement with the programmes. The findings of this qualitative study, along with results from the feasibility trial, will assist in supporting the decision of whether it is feasible to conduct a definitive randomised controlled trial to determine the clinical and cost‐effectiveness of Group e‐rehab and My Knee UK for wider implementation.

## Author Contributions

Funding was secured by Gretl A. McHugh, Philip G. Conaghan, Sarah R. Kingsbury, Kim L. Bennell, Mark Conner and Christine Comer. The Australian SMS Programme and My Knee Exercise were co‐developed by Kim L. Bennell, Rana S. Hinman and Rachel K. Nelligan. The qualitative evaluation was developed by Gretl A. McHugh and data collected by Elizabeth C. Lavender and Dawn Groves‐Williams. Gretl A. McHugh and Elizabeth C. Lavender analysed the data. Gretl A. McHugh draughted the manuscript, and all authors contributed to the review of the manuscript and approved the final version.

## Ethics Statement

West of Scotland Research Ethics Committee 5 (REF: 20/WS/006).

## Conflicts of Interest

The authors declare no conflicts of interest.

## Data Availability

The data that support the findings of this study are available on request from the corresponding author. The data are not publicly available due to privacy or ethical restrictions.

## References

[msc70051-bib-0001] Ackerman, I. N. , J. L. Kemp , K. M. Crossley , A. G. Culvenor , and R. S. Hinman . 2017. “Hip and Knee Osteoarthritis Affects Younger People, Too.” Journal of Orthopaedics & Sports Physical Therapy 47, no. 2: 67–79. 10.2519/jospt.2017.7286. 28142365

[msc70051-bib-0002] Anderson, A. M. , E. C. Lavender , E. Dusabe‐Richards , et al. 2021. “Peer Mentorship to Improve Self‐Management of Hip and Knee Osteoarthritis: A Randomised Feasibility Trial.” BMJ Open 11, no. 7: e045389. 10.1136/bmjopen-2020-045389. PMC8296761 34290063

[msc70051-bib-0003] Bennell, K. L. , R. Nelligan , F. Dobson , et al. 2017. “Effectiveness of an Internet‐Delivered Exercise and Pain‐Coping Skills Training Intervention for Persons With Chronic Knee Pain: A Randomized Trial.” Annals of Internal Medicine 166, no. 7: 453–462. 10.7326/M16-1714. 28241215

[msc70051-bib-0004] Braun, V. , and V. Clark . 2021. Thematic Analysis: A Practical Guide. London: Sage Publication.

[msc70051-bib-0005] Centre for Ageing Better . 2023. Stage of Ageing. https://ageing‐better.org.uk/sites/default/files/2023‐12/The‐State‐of‐Ageing‐interactive‐summary‐2023‐4.pdf?_gl=1*i0l7m9*_up*MQ..*_gs*MQ..&gbraid=0AAAAACS1nFC‐lrrV4_uVu333kXi6RXKjh.

[msc70051-bib-0006] Centre for Policy on Ageing. Rapid Review . 2014. The Potential Impact of New Technologists. http://www.cpa.org.uk/information/reviews/CPA‐Rapid‐Review‐The‐potential‐impact‐of‐new‐technology.pdf.

[msc70051-bib-0007] Chartered Society of Physiotherapy . 2024. MSK Waiting Times and the Physiotherapy Staff Shortage – May in the Media. https://www.csp.org.uk/news/2024‐06‐05‐msk‐waiting‐times‐physiotherapy‐staff‐shortage‐may‐media.

[msc70051-bib-0008] Cui, A. , H. Li , D. Wang , J. Zhong , Y. Chen , and H. Lu . 2020. “Global, Regional Prevalence, Incidence and Risk Factors of Knee Osteoarthritis in Population‐Based Studies.” EClinicalMedicine 26: 29–30. 10.1016/j.eclinm.2020.100587. PMC7704420 34505846

[msc70051-bib-0009] Dobson, F. , K. L. Bennell , S. D. French , et al. 2016. “Barriers and Facilitators to Exercise Participation in People With Hip and/or Knee Osteoarthritis: Synthesis of the Literature Using Behavior Change Theory.” American Journal of Physical Medicine & Rehabilitation 95, no. 5: 372–389. 10.1097/PHM.0000000000000448. 26945211

[msc70051-bib-0010] Doyle, L. , A.‐M. Brady , and G. Byrne . 2016. “An Overview of Mixed Methods Research–Revisited.” Journal of Research in Nursing 21, no. 8: 623–635. 10.1177/1744987116674257.

[msc70051-bib-0011] Doyle, L. , C. McCabe , B. Keogh , A. Brady , and M. McCann . 2020. “An Overview of the Qualitative Descriptive Design Within Nursing Research.” Journal of Research in Nursing 25, no. 5: 443–455. 10.1177/1744987119880234. 34394658 PMC7932381

[msc70051-bib-0012] Fransen, M. , S. McConnell , A. R. Harmer , M. Van der Esch , M. Simic , and K. L. Bennell . 2015. “Exercise for Osteoarthritis of the Knee.” Cochrane Database of Systematic Reviews Issue 1: CD004376. 10.1002/14651858.CD004376.pub3. PMC10094004 25569281

[msc70051-bib-0013] Goff, A. J. , D. De Oliveira Silva , M. Merolli , E. C. Bell , K. M. Crossley , and C. J. Barton . 2021. “Patient Education Improves Pain and Function in People With Knee Osteoarthritis With Better Effects When Combined With Exercise Therapy: A Systematic Review.” Journal of Physiotherapy 67: 177–189. 10.1016/j.phys.2021.06.011. 34158270

[msc70051-bib-0014] Groves‐Williams, D. , E. C. Lavender , C. Comer , et al. 2023. “Developing and Adapting Two Electronic‐Rehabilitation Programmes for Persistent Knee Pain.” Musculoskeletal Care 21, no. 4: 1307–1314. 10.1002/msc.1812. 37622339 PMC10947164

[msc70051-bib-0015] Groves‐Williams, D. , G. A. McHugh , K. L. Bennell , et al. 2022. “Evaluation of Two Electronic‐ Rehabilitation Programmes for Persistent Knee Pain: Protocol for a Randomised Feasibility Trial.” BMJ Open 12, no. 6: e063608. 10.1136/bmjopen-2022-063608. PMC9171213 36194515

[msc70051-bib-0016] Hall, A. K. , H. Cole‐Lewis , and J. M. Bernhardt . 2015. “Mobile Text Messaging for Health – A Systematic Reviews of Reviews.” Annual Review of Public Health 36, no. 1: 393–415. 10.1146/annurev-publhealth-031914122855. PMC4406229 25785892

[msc70051-bib-0017] Hinman, R. S. , P. K. Campbell , A. J. Kimp , et al. 2024. “Telerehabilitation With a Physiotherapist for Chronic Knee Pain Versus In‐Person Consultations in Australia: The PEAK Non‐Inferiority Randomised Controlled Trial.” Lancet 403, no. 10433: 1267–1278. 10.1016/S0140-6736(23)02630-2. 38461844

[msc70051-bib-0018] Hinman, R. S. , S. E. Jones , R. K. Nelligan , et al. 2023. “Absence of Improvement With Exercise in Some Patients With Knee Osteoarthritis: A Qualitative Study of Responders and Nonresponders.” Arthritis Care & Research 75, no. 9: 1925–1938. 10.1002/acr.25085. 36594402

[msc70051-bib-0019] Holden, M. A. , E. E. Nicholls , J. Young , E. M. Hay , and N. E. Foster . 2012. “Role of Exercise for Knee Pain. What Do Older Adults in the Community Think?” Arthritis Care & Research 64, no. 10: 1554–1564. 10.1002/acr.21700. 22511582

[msc70051-bib-0020] King, N. 2004. “Using Templates in the Thematic Analysis of Text.” In Essential Guide to Qualitative Methods in Organizational Research, edited by C. Cassell and G. Symon , 257–270. London, UK: Sage.

[msc70051-bib-0021] Ledingham, A. , E. S. Cohn , K. R. Baker , and J. J. Keysor . 2019. “Exercise Adherence: Beliefs of Adults With Knee Osteoarthritis Over 2 Years.” Physiotherapy Theory and Practice 36, no. 12: 1363–1378. 10.1080/09593985.2019.1566943. 30652930

[msc70051-bib-0022] MacPhail, C. , N. Khoza , L. Abler , and M. Ranganathan . 2016. “Process Guidelines for Establishing Intercoder Reliability in Qualitative Studies.” Qualitative Research 16, no. 2: 198–212. 10.1177/1468794115577012.

[msc70051-bib-0023] Marks, R. , and J. P. Allegrante . 2005. “Chronic Osteoarthritis and Adherence to Exercise: A Review of the Literature.” Journal of Ageing and Physical Activity 13, no. 4: 434–460. 10.1123/japa.13.4.434. 16301755

[msc70051-bib-0024] Mason, S. J. , L. M. Brading , K. Kane , P. G. Conaghan , S. R. Kingsbury , and G. A. McHugh . 2024. “Barriers and Facilitators to Engaging With a Digital Self‐Management Programme for Painful Distal Upper Limb Musculoskeletal Disorders: A Qualitative Exploratory Study.” Health Expectations 27, no. 3: e14056. 10.1111/hex.14056. 38858844 PMC11164711

[msc70051-bib-0025] Mo, L. , B. Jiang , T. Mei , and D. Zhou . 2023. “Exercise Therapy for Knee Osteoarthritis: A Systematic Review and Network Meta‐Analysis.” Orthopaedic Journal of Sports Medicine 11, no. 5: 23259671231172773. 10.1177/23259671231172773. 37346776 PMC10280533

[msc70051-bib-0026] National Institute for Health and Care Excellence . 2022. Osteoarthritis in the Over 16s: Diagnosis and Management. London: NICE. 36745715

[msc70051-bib-0027] Nelligan, R. K. , R. S. Hinman , L. Atkins , and K. L. Bennell . 2019. “A Short Message Service Intervention to Support Adherence to Home‐Based Strengthening Exercise for People With Knee Osteoarthritis: Intervention Design Applying the Behavior Change Wheel.” Journal of Medical Internet Research MHealth and UHealth 7, no. 10: e14619. 10.2196/14619. PMC7012505 31628786

[msc70051-bib-0028] Nelligan, R. K. , R. S. Hinman , J. Kasza , S. J. C. Crofts , and K. L. Bennell . 2021. “Effects of a Self‐Directed Web‐Based Strengthening Exercise and Physical Activity Program Supported by Automated Text Messages for People With Knee Osteoarthritis: A Randomized Clinical Trial.” JAMA Internal Medicine 181, no. 6: 776–785. 10.1001/jamainternmed.2021.0991. 33843948 PMC8042569

[msc70051-bib-0029] Nelligan, R. K. , R. S. Hinman , P. L. Teo , and K. L. Bennell . 2020. “Exploring Attitudes and Experiences of People With Knee Osteoarthritis Toward a Self‐Directed eHealth Intervention to Support Exercise: Qualitative Study.” JMIR Rehabilitation and Assistive Technologies 7, no. 2: e18860. 10.2196/18860. 33242021 PMC7728537

[msc70051-bib-0030] Office for Health Improvement and Disparities . 2023. Obesity Profile: Short Statistical Commentary. https://www.gov.uk/government/statistics/obesity‐profile‐update‐may‐2023/obesity‐profile‐short‐statistical‐commentary‐may‐2023.

[msc70051-bib-0031] Peat, G. , R. McCarney , and P. Croft . 2001. “Knee Pain and Osteoarthritis in Older Adults: A Review of Community Burden and Current Use of Primary Health Care.” Annals of the Rheumatic Diseases 60, no. 2: 91–97. 10.1136/ard.60.2.91. 11156538 PMC1753462

[msc70051-bib-0032] Raposo, F. , M. Ramos , and A. Lúcia Cruz . 2021. “Effects of Exercise on Knee Osteoarthritis: A Systematic Review.” Musculoskeletal Care 19, no. 4: 399–435. 10.1002/msc.1538. 33666347

[msc70051-bib-0033] Rogers, M. A. , K. Lemmen , R. Kramer , J. Mann , and V. Chopra . 2017. “Internet‐Delivered Health Interventions That Work. Systematic Review of Meta‐Analyses and Evaluation of Website Availability.” Journal of Medical Internet Research 19, no. 3: e90. 10.2196/jmir.7111. 28341617 PMC5384996

[msc70051-bib-0034] Sandelowski, M. 2010. “What’s in a Name? Qualitative Description Revisited.” Research in Nursing & Health 33, no. 1: 77–84. 10.1002/nur.20362. 20014004

[msc70051-bib-0035] Stockwell, S. , P. Schofield , A. Fisher , et al. 2019. “Digital Behavior Change Interventions to Promote Physical Activity and/or Reduce Sedentary Behavior in Older Adults: A Systematic Review and Meta‐Analysis.” Experimental Gerontology 120: 68–87. 10.1016/j.exger.2019.02.020. 30836130

[msc70051-bib-0036] Tong, A. , P. Sainsbury , and J. Craig . 2007. “Consolidated Criteria for Reporting Qualitative Research (COREQ): A 32‐Item Checklist for Interviews and Focus Groups.” International Journal for Quality in Health Care 19, no. 6: 349–357. 10.1093/intqhc/mzm042. 17872937

[msc70051-bib-0037] Vasileiou, K. , J. Barnett , S. Thorpe , and T. Young . 2018. “Characterising and Justifying Sample Size Sufficiency in Interview‐Based Studies: Systematic Analysis of Qualitative Health Research Over a 15‐Year Period.” BMC Medical Research Methodology 18, no. 1: 148. 10.1186/s12874-018-0594-7. 30463515 PMC6249736

[msc70051-bib-0038] Webb, J. , H. Allison , and M. Mprah . 2024. “Let’s Move With Leon – A Qualitative Evaluation of a UK Digital Intervention to Improve Physical Activity in People With a Musculoskeletal Condition.” Public Health 227: 32–37. 10.1016/j.puhe.2023.11.09. 38103274

[msc70051-bib-0039] Zeni, J. A. Jr. , M. J. Axe , and L. Snyder‐Mackler . 2010. “Clinical Predictors of Elective Total Joint Replacement in Persons With End‐Stage Knee Osteoarthritis.” BMC Musculoskeletal Disorders 11, no. 1: 86. 10.1186/1471-2474-11-86. 20459622 PMC2877653

